# Understanding viroids, endogenous circular RNAs, and viroid-like RNAs in the context of biogenesis

**DOI:** 10.1371/journal.ppat.1012299

**Published:** 2024-06-27

**Authors:** Jie Hao, Junfei Ma, Ying Wang

**Affiliations:** 1 Department of Plant Pathology, University of Florida, Gainesville, Florida, United States of America; 2 Plant Molecular and Cell Biology Program, University of Florida, Gainesville, Florida, United States of America; University of Iowa, UNITED STATES

## Introduction

**Fig 1:**
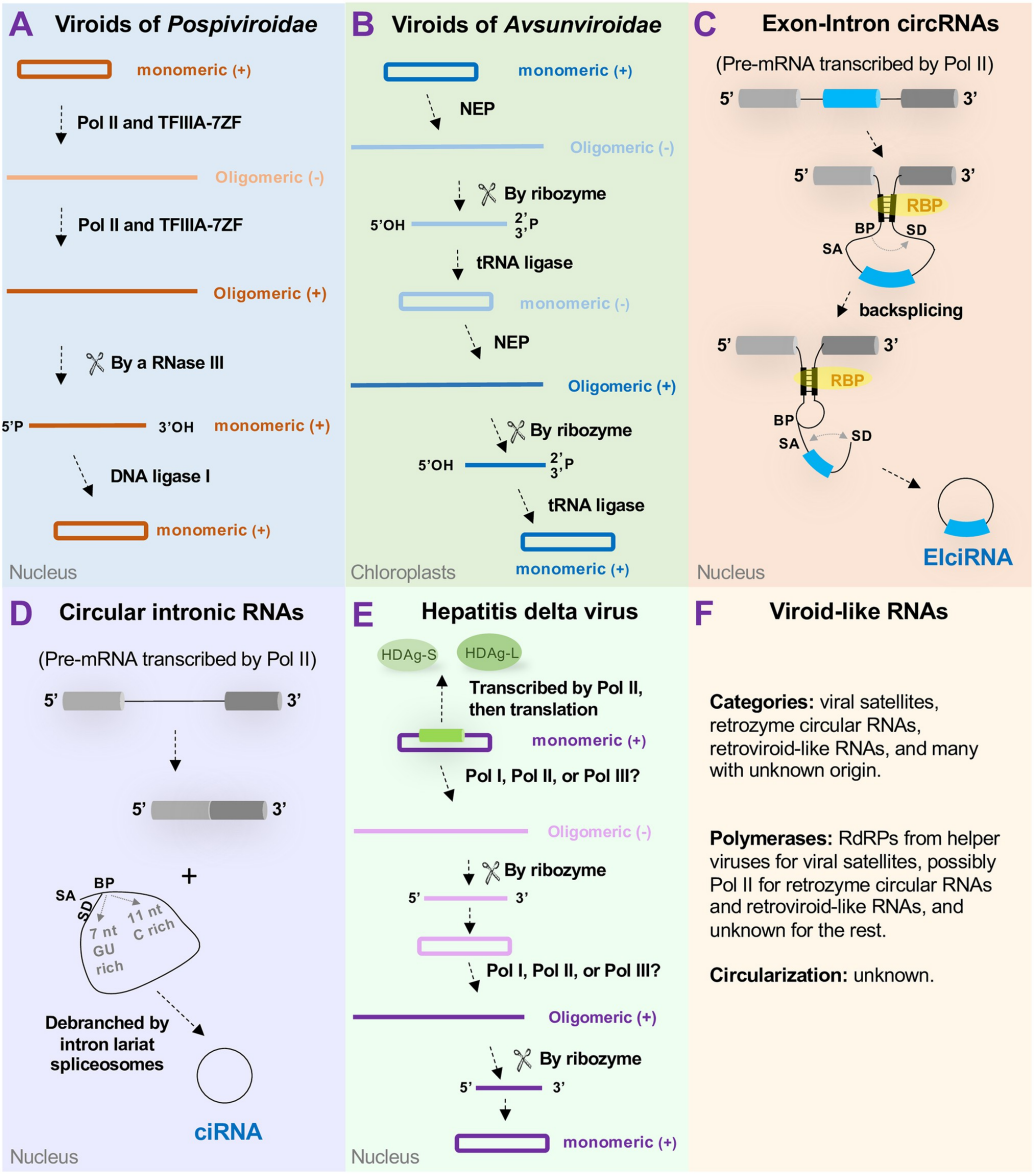
Biogenesis of viroids, circular RNAs, hepatitis delta virus, and viroid-like RNAs. (**A**) *Pospiviroidae*; (**B**) *Avsunviroidae*; (**C**) EIciRNAs; (**D**) ciRNAs; (**E**) hepatitis delta virus; and (**F**) viroid-like RNAs. Note that backsplicing (a downstream 5′ splice donor joined to an upstream 3′ splice acceptor) in (**C**) can be regulated by either self-complementary sequences or RBPs. Pol I, Pol II, and Pol III depict RNA polymerases I, II, and III, correspondingly. TFIIIA-7ZF, transcription factor IIIA splicing isoform with 7 zinc finger domains. In (**C**) and (**D**), cylinders and bars annotate exons, and solid lines depict introns. (**C**) and (**D**) are adapted from reference [39]. BP, branching point; ciRNA, circular intronic RNA; EIciRNA, exon-intron circRNA; HDAg, hepatitis delta antigen; NEP, nuclear-encoded RNA polymerase; RBP, RNA-binding protein; SA, splicing acceptor; SD, splicing donor.

Viroids are a group of noncoding subviral RNAs that infect plant hosts. Currently, there are 44 formal viroid species grouped into 2 families, 39 members in *Pospiviroidae*, and 5 members in *Avsunviroidae* [1]. All viroids share the following characteristics: (1) possessing highly structured circular RNA genomes; (2) lacking protein-coding capacity; (3) lacking DNA intermediates/templates; (4) replicating autonomously without helper virus; (5) using host RNA polymerases that normally recognize DNA templates; and (6) exhibiting transmissibility (Fig 1A and 1B). Interestingly, members of *Avsunviroidae* possess ribozyme activity, which is in contrast to members of *Pospiviroidae*. Viroids were originally considered as pathogens causing crop diseases, but now there are many examples where their infections are symptomless or latent [2]. In general, symptom development depends on the combinations of viroid strains and hosts.

With the rapid development of RNA-Seq technology and bioinformatic tools, an explosive number of endogenous circular RNAs have been identified in nearly all examined organisms across kingdoms. These circular RNAs are encoded by genomes, mostly derived from splicing. Some circular RNAs have been characterized to perform diverse functions [3,4]. More recently, metatranscriptomic analyses have uncovered thousands of RNAs that are predicted to contain the consensus sequence of the hammerhead ribozyme and adopt a circular conformation [5,6]. Due to these similarities to chloroplastic viroids, those sequences are often termed “viroid-like RNAs.” In addition to the well-known viroid analog hepatitis delta virus (HDV) that infects humans [7], some viroid-like RNAs were shown to infect fungal hosts [8]. Therefore, more viroid-like RNA pathogens infecting diverse hosts across kingdoms can be expected. Here, we revisit the establishment of the viroid concept and compare the diverse circular RNA biogenesis mechanisms, which can help to understand novel viroid-like RNA pathogens.

## Question 1: What is the definition of viroid?

In the early 20th century, a “spindling-tuber disease” was observed in Irish potato varieties. It was identified as a symptom of infection, but the causal agent was not revealed until the late 1960s [9]. The pathogenic agent (potato spindle tuber viroid (PSTVd)) was characterized as a free RNA that is much smaller than any viral genome [9]. Given its small size, PSTVd was believed to contain no protein-coding capacity. The noncoding nature of viroids was confirmed in the 1970s (collectively reviewed in [10]) and further validated recently with the latest techniques [11].

In the late 1980s, the rolling circle model was proposed to describe viroid replication [12]. This model is based on the observations that (1) all viroids have a circular RNA genome, (2) longer-than-unit-length (+) and (−) RNA intermediates exist, as well as (3) the (−) RNA intermediate and the (+) circular RNA genome form a duplex containing both double-stranded and single-stranded regions (reviewed in [13]). The presence of (−) circular RNA from members of *Avsunviroidae* but not from members of *Pospiviroidae* further established that the former group replicates via the symmetric rolling circle and the latter group replicates via the asymmetric rolling circle (Fig 1A and 1B) [14].

The term “viroid” is used to describe circular noncoding RNAs of exogenous origin that replicate autonomously in host cells via RNA–RNA rolling circle mechanisms without DNA templates or intermediates. Notably, the term was first used by a geneticist (Professor Edgar Altenburg) to describe the potential relationship among viruses, plasmids, and cancers in the “viroid theory” [15], which is not directly related to the current viroids. The discoverer of the first viroid, Dr. Theodor O. Diener, firstly used the term “viroid” to describe PSTVd and its alike, which has been well accepted since 1972.

## Question 2: How to identify viroids and viroid-like RNAs?

Before the RNA-Seq era, viroids were identified as infectious circular RNAs via a retarded pattern in urea polyacrylamide gel electrophoresis. This method remains the gold standard in current practice, because reverse transcription PCR (RT-PCR) cannot distinguish circular genome from linear intermediates [16], which is important for confirming a successful infection. Since the report of PSTVd in 1971, several RNAs that form circular conformations and possess ribozyme activities, such as viral satellites, retrozyme circular RNAs, and retroviroid-like RNAs, have been described [17–19] (Fig 1F).

A decade ago, a strategy using viral small RNAs to reconstruct circular RNA sequences was established allowing high-throughput identification of viroids and viruses [20]. This approach is especially useful for plant samples due to the robust RNA silencing activity therein. The presence of viral sRNAs also suggests that replication has likely occurred [21]. Lately, metatranscriptomic profiling has greatly expanded the number of viroid-like sequences from hundred to several thousand [5,6]. However, circular RNA prediction algorithm is not 100% accurate and the sample source of the datasets are not always clear. Moreover, most of those sequences await experimental validation for their existence and lack functional implications or information on biogenesis. Nevertheless, there are a few viroid-like RNAs that are infectious and replicate autonomously. A well-known example is HDV, which causes hepatitis D in humans [7]. In addition, a couple of viroid-like RNAs can successfully infect fungal cells [8]. Therefore, more viroid-like entities infecting hosts beyond higher plants can be expected.

## Question 3: How can viroids achieve autonomous replication in a cell?

Viroid infection is a continuous process but can be artificially separated into multiple steps, such as cell entry, organelle import/export, transcription, processing, cell-to-cell, and systemic trafficking [1,22,23]. Due to their noncoding nature, viroids must exploit their RNA structures to utilize host machinery for successful infection.

Unlike mammalian viruses, receptors are not required for viroid to enter cells. Instead, they enter the cytoplasm directly through damaged areas of host cells [1]. Post cell entry, members of *Pospiviroidae* use a C-loop RNA motif to recruit Virp1 protein for nuclear entry via Importin alpha4-based pathway. This nuclear import resists oryzalin- or cytochalasin D-treatment, excluding the involvement of cytoskeleton or cytoskeleton-tethered organelles [1]. In the nucleus, viroids direct RNA polymerase II (Pol II) to accept RNA templates with the aid of a plant-specific splicing isoform of *transcription factor IIIA* (*TFIIIA-7ZF*) (Fig 1A) [24,25]. Interestingly, RPL5 protein, the regulator of *TFIIIA* splicing, is directly targeted by PSTVd, thereby modulating *TFIIIA-7ZF* expression. Transcription on viroid RNA templates does not require general transcription factors (TFs) (TFIIA, TFIIB, TFIIS, etc.), which is likely due to the reorganization of Pol II complex (Fig 1A) [26,27]. DNA ligase I catalyzes the circularization of nuclear-replicating viroids [28].

Chloroplastic viroids use nuclear-encoded polymerase (NEP) for replication (Fig 1B) [29]. Their intrinsic ribozyme activity is used for cleavage of intermediates, which are circularized by tRNA ligase (Fig 1B) [30]. A chloroplastic protein, PARBP33, enhances intrinsic ribozyme activity to facilitate viroid processing [31].

## Question 4: What are the possible factors restricting viroid host tropism?

**Fig 2:**
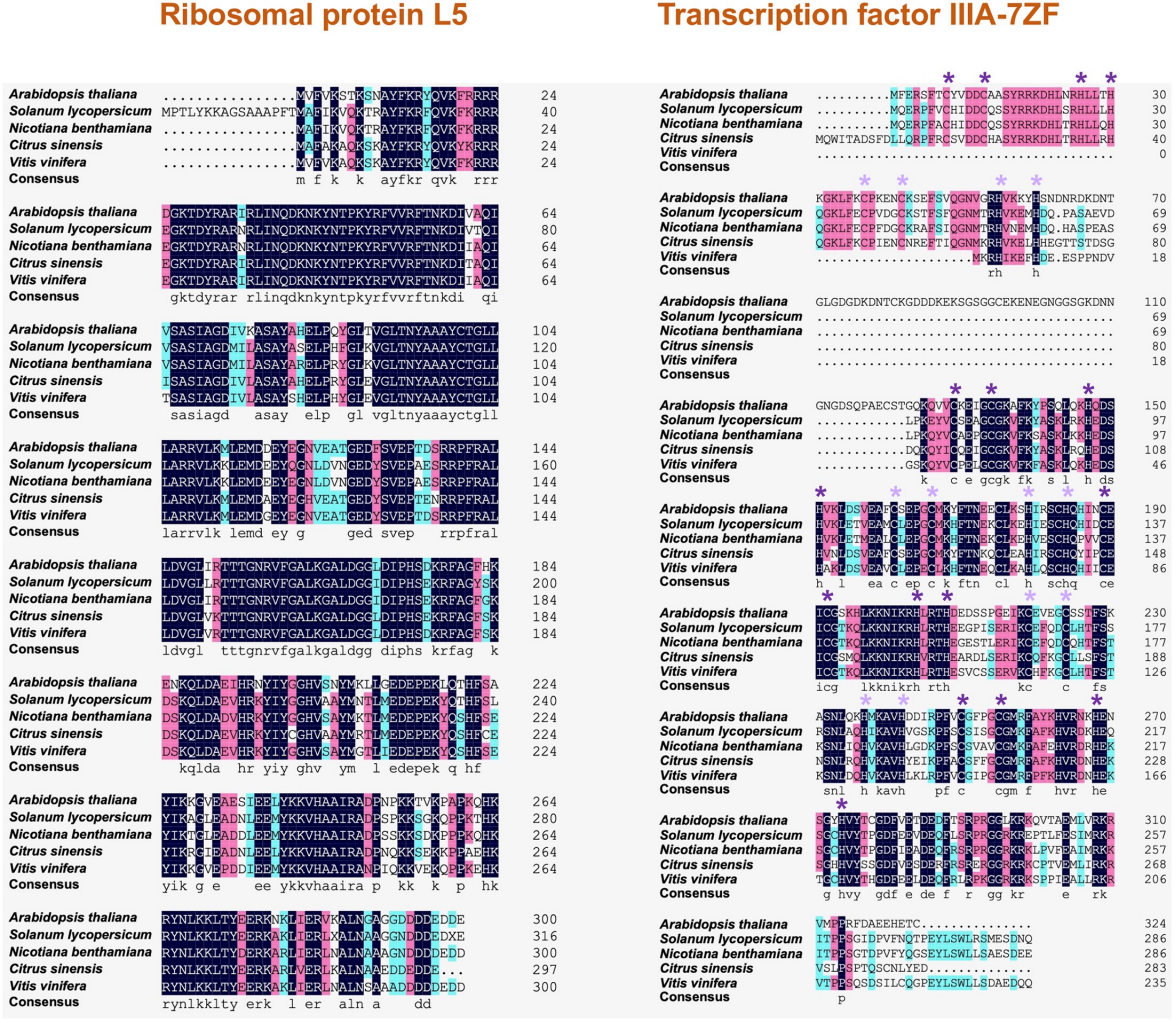
Alignments of 2 viroid host factors from different host plants. Conserved sequences are highlighted by color shades. Cysteine and histidine residues in C2H2 Zinc finger domains (ZF) are highlighted by purple stars. Note that the first 2 ZFs are absent from grape (*Vitis vinifera*).

Viroid RNA motifs, composed of highly arrayed bases via noncanonical pairings in loop regions [32,33], often serve as sites exerting functions, such as determining host tropism. For instance, previous reports have shown that C259U or U257A substitution enables PSTVd variants to infect tobacco [34,35]. Both substitutions occur in the relatively conserved loop E motif, suggesting that loop E modulates host adaptation [36]. The mechanism underlying this phenomenon is unclear, but it is intuitive to reason that RNA motifs should match host factors for binding. For example, TFIIIA-7ZF binds to the lower portion of PSTVd left terminal region [24]. Interestingly, TFIIIA-7ZF is less conserved as compared with another viroid-binding protein RPL5 (Fig 2). Accordingly, the sequences and overall structures of the TFIIIA-7ZF binding region also vary among viroid species [37].

Since viroid replication relies on multiple host factors, some of which are plant specific (for instance, TFIIIA-7ZF), they are not known to replicate in animals. In particular, mammalian cells lack dedicated TF to engage Pol II to RNA templates, so Pol II may not have the necessary processivity to replicate viroids therein. Moreover, evidence suggests that PSTVd can activate dsRNA-activated protein kinases [38], which are a critical component in innate immunity against foreign RNAs.

## Question 5: What are the biogenesis pathways of circular RNAs/viroid-like RNAs?

Recently, a vast number of circular RNAs have been identified. These endogenous circular RNAs are generated from cellular transcripts through the backsplicing mechanism (exon-intron circRNA/EIciRNA and exonic circRNA/ecircRNA) or from splicing lariats that escape from debranching (circular intronic RNA or ciRNA) [39] (Fig 1C and 1D). These lariats are likely converted to true circles by intron lariat spliceosomes [40]. They lack an RNA–RNA amplification process, which is different from viroids. Some functions of circular RNAs include transcriptional regulation in the nucleus as well as regulation on microRNA activities and translation in the cytoplasm [39].

Regarding viroid-like RNAs, HDV, a viroid analog in the mammalian system, replicates via symmetric rolling circle akin to members of *Avsunviroidae* but enters the nucleus and uses Pol II for transcription resembling members of *Pospiviroidae* (Fig 1E). HDV replication may rely on Pol II as well, but there is also evidence suggesting the involvement of Pol I or Pol III [7]. The HDV genome encodes 1 gene but produces 2 proteins (HDAg-L and HDAg-S) due to RNA editing [7]. HDAg-L is responsible for assembly, whereas HDAg-S aids Pol II for transcription [7,41]. HDAg-S and TFIIIA-7ZF, although structurally distinct, exert similar functions in RNA-templated transcription [42].

## Conclusions and perspectives

The explosive discovery of viroid-like sequences, coupled with the functional characterization of a few, has opened a new frontier for the research community: unraveling novel noncoding RNA pathogens in a wider range of hosts beyond plants. However, for most of the viroid-like sequences identified through metatranscriptomic profiling, their existence in cells, biogenesis, and transmissibility await experimental characterization. Knowledge gained from viroid and HDV research, alongside the growing understanding of endogenous circular RNAs, provides a solid foundation for the future exploration of these potentially infectious viroid-like RNAs.
